# A cross-sectional study of the factors associated with male circumcision status among college youth in Ndola, Zambia, 2016

**DOI:** 10.4102/sajhivmed.v20i1.952

**Published:** 2019-06-20

**Authors:** Ernest Kateule, Ramya Kumar, David Mwakazanga, Modest Mulenga, Victor Daka, Gershom Chongwe

**Affiliations:** 1 Zambia Field Epidemiology Training Program, Ministry of Health, Lusaka, Zambia; 2 Tropical Diseases Research Centre, Ndola, Zambia; 3 Zambia AIDS Related Tuberculosis Projects, Lusaka, Zambia; 4 School of Public Health, University of Zambia, Lusaka, Zambia

**Keywords:** College students, Ethnicity, HIV, Voluntary medical male circumcision, Ndola, Zambia

## Abstract

**Background:**

New cases of HIV are increasing among young adults in Zambia; yet voluntary medical male circumcision (VMMC) coverage as an HIV prevention measure remains low. Despite having the highest HIV burden in the province, Ndola district had a VMMC coverage of 23% in 2015 compared to the national target of 80% among high-risk groups.

**Objectives:**

To determine predictive factors associated with circumcision status among male students in Ndola district.

**Methods:**

We conducted a cross-sectional study in May 2016 among students aged 18–35 years enrolled in five conveniently sampled colleges. We administered a structured questionnaire to assess the knowledge, attitudes and perceptions about VMMC. We used multivariable logistic regression to determine factors associated with male circumcision (MC) status.

**Results:**

Of 136 students interviewed, 63% were circumcised, and of those, 96% were medically circumcised. Half of all students were aged 21–24 years. Those who perceived the circumcision procedure to be ‘safe’ (adjusted odds ratio [aOR] = 5.13; 95% CI: 2.09–14.82), and knew that it reduced female to male HIV transmission risk (aOR = 3.65; 95% CI: 3.12–11.67), were more likely to be circumcised. The perception that MC promotes ‘promiscuous behaviour’ (aOR = 0.20; 95% CI: 0.07–0.61), and that sexual sensitivity is the ‘same’ regardless of circumcision status, were associated with not being circumcised (aOR = 0.13; 95% CI: 0.02–0.80).

**Conclusion:**

Students had adequate knowledge about the safety of medical circumcision, and the subsequent risk reduction of HIV infection. Interventions aimed at addressing negative sexual perceptions about circumcision may increase VMMC coverage among college students.

## Introduction

In Zambia, HIV prevalence remains high, with a prevalence of 12.3% and an overall HIV annual incidence among men and women aged 15–49 years of 70 per 10 000 population.^1^ In an effort to combat the high HIV burden, Zambia initiated programmes to expand the provision of voluntary medical male circumcision (VMMC) in 2007.^2^ This policy change was based on evidence from three randomised controlled trials conducted in Kenya, Uganda and South Africa, which showed that male circumcision (MC) reduced the risk of sexual transmission of HIV from women to men by approximately 60%.^3^ An assessment of the potential impact and costs of scaling-up MC in Zambia found that if the government had expanded MC coverage to 80% of all adolescent and adult males by 2015, it would have averted an estimated 486 000 new HIV infections (approximately 50% of all new infections) and would result in substantial cumulative net savings for the public health sector. For these reasons, a national programme to make high-quality and safe MC services available and accessible on a voluntary basis to all HIV-negative men aged 15–49 years was implemented.^4^

Zambia had set an ambitious target of scaling-up VMMC programmes to achieve 80% VMMC coverage by December 2015.^5^ Although there had been significant progress in scaling up high-impact HIV preventive interventions, Zambia fell short of the VMMC coverage goal. The country only managed to circumcise 1 005 424 out of eligible 1 864 396 men during 2012–2015, thus achieving a national VMMC coverage of 54%, instead of the 80% target.^4^ Nevertheless, the new 2016–2020 VMMC operational plan is built on the existing momentum and aims to reach an ambitious target of 90% coverage of men aged 10–49 years, with a focus on those aged 15–29 years. This is also in line with the UNAIDS 90-90-90 strategy to combat the HIV/AIDS pandemic, and the ultimate elimination of new HIV infections by 2030.^6^

The scale-up of VMMC in Zambia is constrained by the low demand for MC services, especially from young men.^3^ Studies conducted in sub-Saharan Africa and other parts of the world have suggested various barriers to seeking VMMC services. Studies have suggested that gaps in knowledge regarding VMMC services, misconception, myths and negative influences from different sources such as the community and families affect the acceptability of VMMC.^7,8,9^ Confusion of VMMC with female genital mutilation, fear of pain, cultural and religious beliefs, cost, the risk of medical complications and adverse effects, and the possibilities that MC would result in increased sexual risk behaviours or behavioural disinhibitions all influence VMMC uptake rates.^7^ Other barriers and risks include the lack of regular access or no access to healthcare at all, expected time away from employment in order to heal, lack of spousal support, a reduction in penile sensitivity and size, and fear of a lessened capacity to engage in sexual intercourse.^10,11,12^

To date, few studies in Zambia have been conducted to determine the MC status and identify predictive factors associated with VMMC uptake among male college youth. A study conducted in one college in Mansa district, a rural part of Zambia, found that respondents ranked enhanced sexual performance and pleasure as the most important reason why they would choose circumcision.^13^ Almost all (97%, *N* = 25) male respondents thought that loss of penile sensitivity was beneficial because it allowed both men and women to enjoy sex for longer periods.^13^ However, this study sampled from a non-circumcising community, and the lack of variability in sociodemographic characteristics among participants, limited the investigators’ ability to make comparisons across traditionally circumcising and non-circumcising communities. Factors associated with VMMC uptake may differ between rural and urban areas. Thus, we conducted a study targeting male college and university students aged 18–35 years in an urban setting in order to determine attitudes and knowledge levels around MC, as well as to explore sociocultural factors that influence young men to elect for VMMC services.

## Methods

### Study design

A cross-sectional survey study was conducted to determine the prevalence and correlates of MC uptake among males aged 18–35 years. This study design was preferred to a mixed-methods study because there was inadequate time to conduct the extensive follow-up required for a cohort study or focus group discussions (FGDs), as students were time-constrained because of full course loads.

### Timeframe

The study was conducted within a duration of 1 month, from 02 to 27 May 2016.

### Population

The targeted population was about 12 710 students in Ndola district. The sample comprised students from Ndola College of Biomedical Sciences, Zambia Information Communication and Technology College, Northern Technical College, Zambia Electricity Supply Company (ZESCO) Training Centre and the Copperbelt University Ndola Campus.

### Setting

Ndola is the third largest city in Zambia, with a population of 451 246. It is the industrial and commercial centre of the Copperbelt, Zambia’s copper-mining region, and capital of Copperbelt Province. Nearly half (49.4%) of the population are men and the majority (223 020; 58.4%) are aged 15–64 years. The youth (15–24 years) represents 23.5% of the urban population, while the overall median age is 18.5 years.

### Sample size and sampling procedure

To estimate the knowledge of VMMC among the students with a 95% confidence level, and assuming a prevalence of 95% ± 4%, using Kirsh’s (1965) method of sample size calculation, the minimum required sample size was 115. We obtained a sample size of 176 after adjusting for a non-response rate of 35%. We assumed uniform design effect across the colleges as the targeted colleges were in the same district and within a 5 km radius of each other.

We introduced the study to the heads of institutions or departments at each of the colleges, who then went on to formally announce the study to their respective student bodies. Students who fulfilled the inclusion criteria (male, aged 18–35 years) were approached in their lecture halls typically at the end of their lectures and asked if they would like to participate in the study. Respondents who met the inclusion criteria were enrolled regardless of the year of study or HIV status. Students who did not consent to participate in the study were excluded. We did not expect differences to occur in characteristics between those who consented to participate in the study and those who refused as only those that fitted the same recruitment criteria were approached for consent. The number of respondents recruited at each study site was obtained using the probability proportional to size approach.

### Data collection

We administered a standardised questionnaire to eligible participants through in-person interviews. The questionnaire was piloted on five students at one college in order to improve the reliability and validity of the questionnaire. The data collection instrument consisted of three sections: the participant’s sociodemographic characteristics, knowledge about VMMC and attitudes towards VMMC as a prevention strategy against HIV transmission. In this study, our outcome variable was the reported MC status among college youth. We collected data on exposure variables such as demographics (age, marital status, religion, year of study and ethnicity/tribe), knowledge (awareness about medical MC services, reduced risk of HIV infection, reduced risk of other sexually transmitted infections [STIs], safety of procedure and penile hygiene) and attitudes or perceptions (access to and costs of MC services, fear of pain, fear of HIV testing, sexual dissatisfaction or satisfaction, promiscuousness, and stigma). The questionnaire was self-developed based on a literature review of various published studies, such as the Zambian Demographic and Health Surveys, as well as unpublished reports.^3^ The 10–15 min interviews were conducted by trained research assistants who were fluent in both English and Bemba, which are the primary local languages. To minimise bias during data collection, we ensured age–sex matching, that is, only male research assistants administered the questionnaire after lecture hours in private rooms identified at each of the five campuses.

### Data management

The paper-based questionnaires were physically checked for completeness and consistency before data were entered into Epi Info^TM^ (Centers for Disease Prevention and Control, Atlanta, GA, US; Version 3.5.1). We used Epi Info to conduct checks on data range, values and consistency. Data were then exported to SPSS version 22.0 for further statistical analyses. For clearer interpretation of results, ordinal variables were recorded to binary form; responses ranging from ‘very important’ to ‘important’ were recoded to ‘yes’, whereas ‘somewhat important’ and ‘not important’ were recoded to ‘no’. The resulting data set included both categorical and continuous variables.

### Statistical analyses

We generated means with their standard deviations to describe continuous variables, and frequency and percentage distributions to describe categorical variables. We used univariable logistic regressions to assess individual statistical significance of association between factors and MC status, and multivariable logistic regression to adjust for other factors. The multivariable logistic regression model was determined in two stages. Firstly, cross tables of the factors associated with VMMC were done; at this point, chi-square test or Fisher’s exact-based *p*-values, as appropriate, equal to or less than 0.10 were deemed indicative of a significant association. Secondly, factors found to be significantly associated with VMMC at the first stage were taken into the multivariable logistic regression model. The Wald chi-square test-based *p*-values equal to or less than 0.05 were deemed indicative of a significant association in the multivariable model.

### Ethical consideration

This study was conducted after approval from ERES Converge Research Ethics Board (Reference number 00005948) was obtained. A written informed consent was obtained after explaining the purpose of the study to prospective participants. This was a low-risk single-contact study using a structured questionnaire and participants were free to withdraw from the study or decline to answer questions any time without consequences. The interviews were conducted in a private environment.

## Results

Of the total target sample of 176 male participants, 136 consented, with a response rate of 77.2%. The majority were aged between 21 and 24 years (50.0%). Of the participants interviewed, 28% were from Northern Technical College, followed by the Copperbelt University (24.4%) and Zambia ICT College (12.6%). Most of the respondents reported that they were not in committed relationships (85.1%), although half reported having sexual partners (50.8%). The majority reported identifying with traditionally non-circumcising cultural groups (82.1%), such as Bemba, Lozi, Tonga and Ngoni (Table 1). The overwhelming majority of study participants identified as Christian (99.2%, *n* = 133), a religion that does not generally promote circumcision on religious grounds.

**TABLE 1:** Demographic characteristics, circumcision status and awareness about medical male circumcision services (*N* = 136).

Characteristics	*n*	%
**How old are you? *n* = 135**		
17–20 years	37	27.1
21–24 years	68	50.0
25–28 years	17	12.7
29+ years	14	10.2
**What is your marital status? *n* = 134**		
Single	114	85.1
Married	12	8.9
Cohabiting	8	6.0
**Study site (*n* = 136)**		
ZESCO Training Centre	22	16.3
Northern Technical College	38	28.1
Biomedical Science College	25	18.5
Zambia ICT College	17	12.6
Copperbelt University	33	24.4
**Which religious denomination do you belong to? (*n* = 136)**		
Christian	132	97.0
Muslim	1	0.7
None	2	1.5
**Are you circumcised? (*n* = 131)**		
Yes	82	62.6
No	44	33.6
No response	5	3.8
**Which circumcision type did you undergo? (*n* = 82)**		
Medical	78	96.3
Traditional	3	3.7
**Are you considering to get circumcised in future? (*n* = 44)**		
Yes	38	74.5
No	8	15.7
Don’t know	5	9.8
**Should VMMC be promoted in non-circumcising communities? (*n* = 136)**		
Yes	123	90.4
No	3	2.2
Don’t know	8	5.9
**Have you ever seen or heard messages on VMMC?**		
Yes	130	97.7
No	3	2.3
**Do you know of a place where VMMC procedures are done?**		
Yes	125	91.9
No	4	3.0
Missing	7	5.1
**Are male circumcision services provided for free in public hospitals or clinics? (*n* = 136)**		
Yes	122	39.7
No	4	2.9
Don’t know	10	7.3
**Where are VMMC procedures done? (multiple responses)**		
Government hospital	110	88.0
Government clinic	79	63.2
Private hospital	34	27.6
Private hospital	27	21.6
Other (NGOs, mobile services)	3	2.4
**Describe how easy or difficult is it to get VMMC site(s)**		
Easily accessed	91	70.0
Accessed with difficulty	25	18.4
Don’t know	5	3.7
Missing	11	8.1
**How often are VMMC services available?**		
Daily (7 days a week)	41	30.1
At least twice in a week	18	13.2
Weekdays (Monday to Friday)	35	25.7
Don’t know	27	19.9
Missing	11	8.1

NGO, non-governmental organisation; VMMC, voluntary medical male circumcision; ZESCO, Zambia Electricity Supply Company.

Of the 131 students who responded to a question about their circumcision status, 82 (62.6%) reported being circumcised. The majority (81.5%) of the circumcised students reported being single. The vast majority of medically circumcised respondents believed that VMMC should be promoted in non-circumcising communities (98.6%), and 74.5% of uncircumcised respondents reported that they were considering circumcision in the future. Regardless of circumcision status, 97.6% (*n* = 126) of respondents recommended MC as an additional strategy to prevent HIV and other STIs.

Awareness of VMMC was almost universal, with 97.7% (*n* = 133) of the respondents reporting having seen or heard messages about it (Table 1). Many respondents ranked government hospitals and clinics among the top two places where one could get circumcised (88.0% and 63.2%, respectively), and the majority knew that the services at those locations were free (96.8%). Most respondents reported that they were able to easily access VMMC sites (75.2%, *n* = 131). About one in five (22.3%, *n* = 121) did not know what time during the day these services were provided (Table 1).

With regard to knowledge and attitude towards VMMC, circumcised participants ranked the following as reasons, in order of importance, why they decided to get circumcised: (1) it reduces the chances of contracting HIV (90%, *n* = 80), (2) it reduces the chances of contracting other STIs (88.8%, *n* = 80), (3) it improves penile hygiene (93.2%, *n* = 74), (4) these services were provided for free (87.3%, *n* = 79), and (5) facilities were nearer to their residences (87.2%, *n* = 78) (Table 2a and Table 2b). Other respondents reported that MC reduces the risk of developing penile cancer (90.0%), while some reported that the services were safe (96.2%) and of good quality (90.4%). Nearly two-thirds (*n* = 80) of students ranked enhanced sexual pleasure as an important reason for accepting the procedure. Eight exposure variables (such as the safety of procedure, costs, the quality of MC services, perceptions on promiscuous behaviour, sexual enhancement, sexual sensitivity being the ‘same’ regardless of circumcision status, reduced chance of HIV and other STIs) met the level of statistical significance in the univariate model and were then taken into the multivariate model (Table 3).

**TABLE 2a:** Exposure variables for male circumcision status among college youth, Ndola – 2016 (*N* = 136).

Variable	Response			
	*n*	Agree	Disagree	%
**What were the important reasons as to why you decided to get circumcised?**† **(*n* = 82)**				
MC reduces the chances of HIV	80	72	8	90.0
MC reduces the chances of STIs	80	71	9	88.8
MC improves penile hygiene	74	69	5	93.2
MC services are provided for free	79	69	10	87.3
MC facilities are near where I live	78	68	10	87.2
MC reduce chances of penile cancer	80	72	8	90.0
MC services are of good quality	79	76	3	96.2
MC is a safe procedure	73	66	7	90.4
MC enhances sexual pleasure	80	52	28	65.0
**What are the important reasons as to why you are not circumcised? (*n* = 44)**				
Concerned about MC complication	40	28	12	70.0
MC does not guarantee 100% protect against STI/HIV	37	23	14	62.2
Fear of pain	42	24	18	57.1
Six weeks post-surgery abstinence	40	25	15	62.5
Long healing after MC	39	25	14	64.1
MC is not a safe procedure	38	18	20	47.4
Fear of injections	40	18	22	45.0
MC promotes promiscuity	40	19	21	47.5

MC, male circumcision; STI, sexually transmitted infection; OR, odds ratio; CI, confidence interval; aOR, adjusted odds ratio.

*n* = number who consented or responded to the question (variable); the valid counts for analyses (%).

† , Reasons for MC, *n* = 82; Not Applicable (N/A) = 54; Missing = (82-*n*′) per each variable.

**TABLE 2b:** Exposure variables for male circumcision status among college youth, Ndola – 2016 (*N* = 136).

Variable	Response					
	OR	95% CI	*p*	aOR	95% CI	*p*
**Factors associated with circumcision status among male college students (*N* = 136)**						
MC reduces chances of contracting HIV	4.48	1.26–15.94	0.01	5.13	2.09–14.82	< 0.01
MC is a safe procedure/surgery	4.05	1.12–14.80	0.05	3.65	3.12–11.67	< 0.01
Circumcised and uncircumcised are same	0.19	0.04–0.86	0.03	0.13	0.02–0.80	0.02
MC promotes promiscuous behaviour	0.23	0.09–0.57	< 0.01	0.20	0.07–0.61	< 0.01

MC, male circumcision; STI, sexually transmitted infection; OR, odds ratio; CI, confidence interval; aOR, adjusted odds ratio.

*n* = number who consented or responded to the question (variable); the valid counts for analyses (%).

**TABLE 3:** Factors associated with circumcision status among male college students, Ndola, Zambia (*N* = 136).

Factors	MC Status				OR	95% CI	*p*
	Yes		No				
	*n*	%	*n*	%			
**MC reduces chances of contracting HIV**							
Yes	74	94.9	33	80.5	4.49	1.26–15.95	0.03
No	4	5.1	8	19.5	1	1	-
**MC is a safe procedure**							
Yes	74	94.9	32	82.1	4.05	1.12–14.80	0.05
No	4	5.1	7	17.9	1	1	-
**Women prefer circumcised men over uncircumcised men**							
Yes	53	68.0	18	46.2	2.47	1.12–5.44	0.04
No	25	32.0	21	53.8	1	1	-
**MC is an expensive procedure**							
Yes	28	36.4	4	10.8	4.71	1.52–14.70	< 0.01
No	49	63.6	33	89.2	1	1	-
**Transport to MC facility is costly**							
Yes	26	33.3	5	13.5	3.20	1.12–9.18	0.05
No	52	66.7	32	86.5	1	1	-
**Concerned about the quality of MC services**							
Yes	39	50.7	10	27.8	2.67	1.14–6.28	0.04
No	38	49.3	26	72.2	1	1	-
**MC promotes promiscuous behaviour**							
Yes	12	16.2	16	45.7	0.23	0.09–0.57	< 0.01
No	62	83.8	19	54.3	1	1	-
**Uncircumcised and circumcised men are the same**							
Yes	22	59.5	28	53.9	0.19	0.04–0.86	0.03
No	55	71.4	24	46.1	1	1	-

MC, male circumcision; OR, odds ratio; CI, confidence interval.

Uncircumcised respondents ranked the following, in order of importance, as reasons why they decided not to get circumcised: (1) surgery complications or safety of MC (70.0%, *n* = 40), (2) MC does not guarantee 100% protection against HIV and other STIs (62.2, *n* = 37), (3) pain during and after surgery (57.1%, *n* = 42), (4) post-surgery abstinence from sex for 6 weeks (62.5%, *n* = 40), and (5) long healing time after MC (64.1, *n* = 39) (Table 2a and Table 2b).

Having the knowledge that MC reduces the risk of female to male HIV transmission (aOR = 3.65; 95% CI: 3.12–11.67) and the belief that the medical procedure is safe (aOR = 5.13; 95% CI: 2.09–14.82) were two statistically significant factors that were associated with being circumcised after controlling for all other factors (Table 3). The perceptions that MC promotes promiscuous behaviour (aOR = 0.20; 95% CI: 0.07–0.61) and that sexual sensitivity is the same for both circumcised and uncircumcised men (aOR = 0.13; 95% CI: 0.02–0.80) were both associated with being uncircumcised after controlling for all other factors.

## Discussion

Of the total 136 students who consented to participate in the study, 63.0% were circumcised, and of those, 96.0% were medically circumcised. Half of all students were aged 21–24 years. Nearly all the students (97.7%) were aware about medical MC and the majority (96.8%) knew that MC services in government health facilities were free. Those who perceived the circumcision procedure to be ‘safe’ and knew that it reduced female to male HIV transmission risk were more likely to be circumcised. The perceptions that MC promotes ‘promiscuous behaviour’ and that sexual sensitivity is the ‘same’ regardless of circumcision status were associated with not being circumcised.

One encouraging finding of this study was that knowledge about VMMC was higher compared to studies conducted in Botswana, Tanzania and Zimbabwe.^10,12,14^ However, the observed differences in levels of VMMC acceptability in other studies could be explained by the diverse cultural settings.^15^ In addition, participants from higher learning institutions are more likely to be exposed to information on VMMC. Our study further confirms the assertion from previous reports that males with more knowledge about benefits and risks of VMMC services are more likely to accept circumcision compared to those with less knowledge.^13,16,17^ Therefore, targeting information, education and communication (IEC) materials about VMMC services to college or university students would further encourage adolescents and young men to take up MC. Additionally, we found that the college population was generally young, single or in uncommitted relationships. This could be explained by the fact that at the time of interviews, we did not find older age groups doing full-time block lectures. Most students aged 30 years and older attend school part-time because they are also engaged in full-time employment. Therefore, the study could not establish the proportion of the males aged above 35 years; hence, further studies may be required to ascertain this age group’s views around MC.

This study found that respondents who had knowledge of the partial protective effect of MC against female to male HIV transmission were more likely to be medically circumcised. It follows that gaps in knowledge of the protective effect of MC against HIV and other STIs could be one reason for the low VMMC uptake among uncircumcised HIV-negative, young males. Similarly, a study in Swaziland documented that the reduced risk of HIV infection was the most important reason for undergoing VMMC (55.4%), followed by reduced risk of other STIs (43.5%) and improved genital hygiene (21.1%).^16^ Similar studies have shown that having the knowledge that ‘VMMC reduces the risk of getting STIs’ had a significant effect on the acceptability of VMMC.^13,16,17^ Uncircumcised respondents who had this knowledge were found to be four times more likely to accept VMMC compared to their counterparts.^16^ Therefore, interventions to increase the awareness of reduced risk of HIV infection may serve to motivate men to undertake VMMC.

Previous studies have documented a strong association between perceptions of safety and VMMC service uptake. Similarly, the ‘cost and quality’ of VMMC services are among well-described factors associated with acceptance levels of medical circumcision in the literature.^10,12,16,18^ In our study, the perceived costs of the procedure had no bearing on respondents’ MC-seeking behaviours. This finding contrasts with a previous study conducted in Zambia, which found that the majority of uncircumcised participants reported that unacceptable medical service conditions and concerns about the quality at VMMC sites were reasons why they did not get circumcised.^15^

Believing that the MC procedure is safe was associated with being medically circumcised. Because this is a cross-sectional study, we cannot say what the temporal relationship is between believing that the MC procedure is safe and actually undergoing VMMC. Other studies conducted in Kenya, Uganda and Zimbabwe found that if men and their parents believed that circumcision leads to high rates of complications, then uptake is likely to be low.^9,11,12,14,17^ For instance, it was noted that parents of uncircumcised boys would be willing to take their sons for circumcision only after the assurance of good treatment and safety of the procedure.^10^

The safety of the procedure is of critical importance to a man’s decision to undergo MC because it is a permanent surgery with potentially significant effects on an individual’s sexual health. Thus, men’s fears about the safety and quality of the procedure should be acknowledged and reassured. The Zambian Ministry of Health along with public health partners should continue to educate individuals that safe, high-quality VMMC services are freely available in government health facilities. More sensitisation on the safety of procedure could correct the negative perceptions around free MC being of poor medical quality.^5,18^ Men considering MC should also be informed about how the procedure is done, what they need to do to prepare for the surgery and how to ensure proper healing post-surgery.

Circumcision is not traditionally practised in the majority of Zambian tribes, which in turn influences societal perceptions and an individual’s decision to seek MC. Studies have documented that men from ethnic communities that do not traditionally circumcise expressed strong feelings against MC. This is because MC is not part of their culture, and they did not want to adopt a practice that their forefathers never had.^10,19^ This could explain the finding in our study that some students perceive circumcision to be an ‘unnatural’ modification of the body organ. It can be expected however that such attitudes may perhaps change gradually with access to accurate information about MC and its benefits.^19,20^

Our study found that some students from non-circumcising tribes were not concerned about their MC status because they believed that both circumcised and uncircumcised men are the same in all aspects, including sexual sensitivity. It is possible that the self-reported preference to undergo MC among circumcised students was because of its protective effect against HIV, and not necessarily whether the respondent’s tribe traditionally practised circumcision or not.^10,19^ Therefore, VMMC programmes should reach out to tribal elders within non-circumcising communities in order to combat some of the negative cultural perceptions and beliefs about VMMC, which could be preventing some men from undergoing MC.

The possibility of suspicion in sexual relationships was reported by some participants in this study. Specifically, uncircumcised men believed that if they underwent circumcision, their partners would perceive them as being more likely to engage in multiple sexual relationships because of the protective effect of MC against STIs including HIV. This phenomenon is known as ‘behavioural disinhibition’.^10^

A study in Zambia noted that almost all male respondents thought that loss of penile sensitivity was beneficial because it allowed both men and women to enjoy sex for longer periods.^10,13^ The reported enhanced sexual satisfaction in men, however, could explain the belief that women have a preference for circumcised men, hence the perception that MC promotes promiscuous behaviour among circumcised males.^9,19,21^ A future study should be conducted to assess Zambian women’s perceptions of VMMC for their spouses and sons. A study among Kenyan urban women aged 18–35 years found that women who were exposed to positive VMMC messages were able to discuss the procedure with their partners, while others made a joint decision for the men to go for VMMC.^22^ This suggests that VMMC programmes could involve women as positive motivators for circumcision, especially because of the indirect benefits to women, including reduced risk of human papillomavirus infection, which is a strong risk factor for cervical cancer.

### Study limitations

In this study, information bias was a possible limitation because the circumcision status was self-reported, and respondents might not have reported their true circumcision status for various reasons. The use of convenient sampling procedures could limit the study’s capacity to appropriately estimate the prevalence of MC among respondents. The study was conducted among college youth, thereby limiting the generalisability to the general population because older age groups may hold different opinions about VMMC. As our sample was restricted to men, we could not capture women’s opinions and were thus unable to make inferences about the role of women in VMMC uptake. However, these anticipated biases were handled by ensuring confidential interviews with male research assistants, and the use of a ‘proportionate to size’ sampling approach to enrol an adequate number of respondents from each of the five campuses. Given the high response rate (77%), our study therefore provides important evidence on the factors associated with MC status among college students in Ndola, Zambia, and provides essential information for programme implementers to effect policy adjustments. The study also offers some baseline information, which is necessary for future research on MC using other contextual approaches.

## Conclusion

Consistent with other studies in African nations, this study showed that most male college students in Ndola district of Zambia had knowledge about the benefits of MC. Believing that the circumcision procedure was safe and that it had a short healing time were factors associated with accepting VMMC services among respondents. The perception that MC promotes promiscuous behaviour, and that sexual sensitivity in circumcised and uncircumcised men is the same were two factors associated with men who self-reported being uncircumcised. Although our study found the level of MC among college students to be higher than the national prevalence, there is a need for the Ministry of Health and its implementing partners to create more awareness about the safety and quality of services in government health facilities. In addition, educating the public that MC reduces the female to male transmission risk of HIV could lead to an increased uptake of VMMC services. There is also a need to carry out similar studies among non-college educated individuals, including females, to determine the knowledge and perceptions about VMMC among both men and women with lower education levels.
